# Which Patients Need an Axillary Clearance after Sentinel Node Biopsy?

**DOI:** 10.4061/2011/195892

**Published:** 2011-08-24

**Authors:** Anastasia Pazaiti, Ian S. Fentiman

**Affiliations:** Research Oncology, Guy's Hospital, 3rd Floor Bermondsey Wing, London SE1 9RT, UK

## Abstract

Sentinel lymph node biopsy (SLNB) is a safe and accurate minimally invasive method for detecting axillary lymph node (ALN) involvement in the clinically negative axilla thereby reducing morbidity in patients who avoid unnecessary axillary lymph node dissection (ALND). Although current guidelines recommend completion ALND when macro- and micrometastatic diseases are identified by SLNB, the benefit of this surgical intervention is under debate. Additionally, the management of the axilla in the presence of isolated tumour cells (ITCs) in SLNB is questioned. Particularly controversial is the prognostic significance of minimal SLNB metastasis in relation to local recurrence and overall survival. Preliminary results of the recently published Z0011 trial suggest similar outcomes after SNB or ALND when the SN is positive, but this finding has to be interpreted with caution.

## 1. Introduction

For patients with operable breast cancer, the major prognostic determinant is whether there has or has not been spread to the axilla and the number of involved axillary nodes [1]. Several theories exist concerning the mechanism of breast cancer cell invasion and metastasis. Initially it was suggested that breast cancer first spreads locoregionally via lymphatics to the axillary lymph nodes and then metastasises more distantly. In accordance with this concept, Halsted developed radical mastectomy as the gold standard for breast cancer surgery [2]. 

Subsequently Fisher postulated that the extent of micrometastases at diagnosis of breast cancer is an indicator of outcome, with biological behaviour of cancer predetermining the likelihood of progression of the disease [3]. Nowadays gene expression profiling arrays can delineate tumour types with different prognoses [4]. The surgical approach for breast cancer treatment evolved from the extensive radical mastectomy and the Patey modified radical mastectomy [5] to breast conserving and minimally invasive techniques [6].

Traditionally the surgical management of breast cancer comprised wide local resection of the primary tumour and axillary lymph node dissection (ALND). Axillary status is the most important prognostic factor in breast cancer providing staging information and therefore largely defining treatment strategy [7]. Diagnostic imaging modalities such as ultrasound, magnetic resonance mammography, positron emission tomography, and 99 m Technetium (Tc) sestamibi scintimammography are not reliable for staging the axilla, particularly with lymph node metastases <0.5 cm [8, 9].

Clinical, pathological, and molecular features are inadequate for assessing ALN metastases. Clinically palpable lymph nodes prove to be false positive in 25–30% of patients [10] and about 40% have positive results after ultrasound with or without fine-needle aspiration node negativity [11]. Tumour size cannot serve as an accurate prognostic indicator for lymph node involvement. Studying 24,740 women with invasive breast cancer, Carter et al. showed that approximately 80% with tumour size <1 cm, 50% with up to 5 cm, and 30% with >5% had negative axilla, a fact suggesting that metastases do not occur exclusively via the axillary lymph nodes, but rather lymph node status serves as an indicator of the tumour's ability to spread [12]. Additionally, it has been recently shown that the molecular profile of the primary tumour is a more significant prognostic indicator in terms of disease-free survival (DFS) and overall survival (OS) than lymph node metastases [4].

The combination of the introduction of population-based mammographic screening for breast cancer, modern imaging methods, and increased public awareness resulted in patients being diagnosed more often with smaller-size tumours and less likelihood of axillary lymph node metastases [13]. It is evident now that 60–70% of patients with early breast cancer are node negative at the time of diagnosis [14] and ALND puts them at significant risk of short- and long-term morbidities without benefit [15]. ALND is associated with acute complication rates of 20–30% including seroma formation, local swelling, numbness, impaired shoulder movement, neuropathy, infection, and chronic lymphoedema rates of 7–37% [16]. In a prospective study by Petrek et al. evaluating a cohort of 923 women with 20 years follow up, it was shown that breast-cancer-related lymphoedema following ALND occurred maximally in the first 3 years following surgery; however, up to 23% of patients may still develop arm swelling during the rest of their lives [17].

Several randomised studies have established that sentinel node biopsy (SNB) is a safe and accurate procedure for detecting tumour cells in SLN and predicting the status of the other axillary nodes (non-SLN). Although accuracy and appropriateness of SNB were disputed by the finding of 5–10% false-negative cases when SNB was followed by axillary dissection at high-risk patients for axillary nodal disease [13, 18], false-negative SNB results seem to have decreased with the increasing experience of surgeons, and it is expected that the utilisation of SNB in the future will be increased [19].

A meta-analysis of seven prospective randomised controlled trials by Kell et al. demonstrated that SNB is equivalent to ALND for the detection of lymph node metastasis with the additional advantage of reduction of up to 75% in morbidity in patients with early stage breast cancer. Furthermore, a trend towards an improved detection of LN metastases was shown when SNB is used [20]. Patients undergoing SNB have a 22% higher odds ratio of having a positive SLN, due to the more intensive pathological examination which utilises multiple sections and immunohistochemistry (IHC) [21]. In contrast, the false-negative cases seen after axillary dissection are probably due to the inability of the pathologist to perform serial sections and IHC on the 20–30 lymph nodes found in a complete axillary clearance specimen.

Studies have shown that SLN is the only positive lymph node in 38–67% of patients when ALNC followed [22]. Interestingly, it has been reported that only 4–8% of patients with negative ALNs have internal mammary lymph node involvement (IMN) whereas 25–50% of patients with affected ALNs have also IMN metastases [23, 24]. Dissection of IMN is not recommended because of the high morbidity and the uncertain benefit on survival [24].

The recently published outcomes of NSABP B-32 trial established the efficacy of SLN biopsy alone with no further ALND in 5611 breast cancer patients with clinically negative lymph nodes [25]. Women with invasive breast cancer who were randomly assigned to either SLN resection plus ALND (group 1) or to SLN resection alone with ALND only if the SLNs were positive (group 2), after 8 years of followup, showed statistically equivalent overall survival, disease-free survival, and regional control. Patient followup is still continuing for longer-term assessment of survival and regional control.

Moreover, a closer look into mature studies focused on axillary relapses and overall survival is in agreement with current findings favouring SLB. The National Surgical Adjuvant Breast and Bowel Project (NSABP) B04 randomised study compared breast cancer patients with clinically negative ALNs managed either by radical mastectomy, total mastectomy with axillary radiation, or total mastectomy alone. The results clearly defined that ALND decreases the risk of locoregional relaps; however, no significant differences in survival were found among the treatment groups [26]. This study has been criticised because of the variability of numbers of lymph nodes resected in the total mastectomy alone arm and the lack of statistical power to detect a small difference in outcome [27]. Indeed, a meta-analysis has suggested that inadequate axillary treatment may lead to not only an increased risk of local relapse but also a 5% reduction in survival [28].

As a result of increasing detection of early breast cancer and the high rate of micrometastases and ITCs (ITCs) found in the detailed pathological examination of SLN, a new debate has opened about the consequent necessity of ALND in these patients. This has arisen because of better understanding of breast cancer behaviour and improved efficacy of combined therapeutic modalities. In this paper we report the current guidelines concerning the management of the axilla after SLNB and review the different aspects arising from recent studies on the role of micrometastases and ITC clusters in SLN on decision making.

## 2. Current Guidelines 

The American Society of Clinical Oncology (ASCO) Expert Panel conducted a systematic review of the literature available through February 2004 on the use of SNB in early-stage breast cancer in order to develop guidelines for the management of the axilla (http://jop.ascopubs.org/content/1/4/134), and these are similar to those of the National Institute of Health and Clinical Excellence (NICE) recommendations in UK (http://www.nice.org.uk/nicemedia/live/12132/43413/43413.pdf) [29]. 

SLNB is recommended for staging patients with clinically negative lymph nodes. ALND is the standard of care in those with a macrometastatic or micrometastatic positive SLN to maximise local control [30]. If the SLN is negative, a cALND (cALND) is not necessary. ITCs detected by IHC are of unknown clinical significance, and when identified, the SLN is regarded as negative and no further ALND is required. Although IHC is often used, it is not included in routine SLN evaluation for breast cancer at this time. ASCO and NICE recommendations for SLNB, ALND alone and managing of the axilla after SLNB are summarised in Table 1. In contrast the German guidelines do not recommend axillary clearance for 1-2 SLN positive in patients with T1 and T2 tumours (http://www.ago-online.de). 

It has been suggested that SLNB should be carried out by an experienced team in order to minimise false negativity and improve the predictive value of the procedure [30]. All suspicious palpable nodes should also be considered as SLNs.

## 3. Micrometastases and Isolated Tumour Cells (ITCs)

The American Joint Committee on Cancer (AJCC) in the sixth edition of the Cancer Staging Manual defined a lymph node metastatic tumour with maximum diameter >2 mm as macrometastasis (pN1), when the diameter of deposit is 0.2–2 mm as micrometastasis (pNmi), and a lesion of single tumour cells or small cell clusters with diameter <0.2 mm as ITCs [pN0(i+)] [31]. ITCs are not distinguishable by H&E staining but detected only with immunohistochemistry (IHC) or molecular methods. Moore et al. suggested that the presence of ITCs was unrelated to known prognostic variables and partly the result of instrumentation and manipulation of the tumour [32].

The management of patients with minimal SLN involvement is problematic [33]. In a meta-analysis of 25 studies of patients with SLN micrometastases, in approximately 20% there was nonsentinel node disease falling to 9% when the SLN involvement was detected by IHC [34]. Furthermore, the consequent effect on DFS and OS remains controversial, so the biological relevance and clinical significance is a matter of debate [35]. 

AMAROS investigates the benefit of a cALND in comparison to treatment with axillary radiotherapy (ART) in patients with SLN-positive breast cancer [36]. A recently published substudy evaluated the identification rate and the nodal involvement of the first 2,000 patients between 2001 and 2005 who entered from 26 European institutions [36]. The sentinel node identification rate was 97% which is high considering the relatively early days of this procedure. 34% were SLN positive of whom 63% had macrometastases, 25% had micrometastases, and 12% had ITCs. In the cALND arm non-SLN involvement was identified in 41% of patients with macrometastases and in 18% of patients with either micrometastases or ITCs.

Several studies have investigated the significance of occult metastases, such as micrometastases or small clusters of tumour cells in association with non-SLN involvement and the impact of cALND on disease-free survival and overall survival, and the larger ones are summarised in Table 2 [37–43]. Although the majority show no prognostic impact of ITCs in the sentinel node, a large Dutch investigation with 5-year followup indicated that women with ITCs who received adjuvant chemotherapy had a significantly better event-free survival compared with untreated cases with ITCs [39]. Furthermore, a Finnish study showed a worse 5-year breast-cancer-specific survival for those with ITC compared with node negative cases [42].

In the largest published multicenter retrospective study of 187 SLN-ITCs patients undergoing cALND, Houvenaeghel et al. reported an incidence of 16% non-SLN involvement [44]. The difference in the risk of non-SLN involvement between sentinel nodes with ITCs (16%) and those with micrometastases (14%) was not statistically significant. However it was not apparent whether the presence of non-SLN metastases should affect the therapeutic decision in these patients. The authors proposed that cALND could be avoided in patients with tubular, colloid, or medullary small primary tumours (pT1) with a risk of non-SLN involvement approximately ≤5%.

In contrast to the conclusions of the aforementioned studies come the MIRROR trial results [45]. MIRROR is a large Dutch cohort retrospective study which assesses the impact of SLN-ITCs and micrometastases on 5-year disease-free survival in patients with favourable primary tumour characteristics. According to recent published data, both patients with SNB micrometastases and those with ITCs who did not undergo cALND experienced a far higher 5-year axillary recurrence rate, 6% in comparison to 1% of SNB-negative patients who did not undergo cALND. Additionally, both patients with SLN micrometastases and ITCs had approximately 5-year disease-free survival improved by 10% with adjuvant systemic therapy. It is important to mention that micrometastases and ITCs had comparable prognostic impact [46]. MIRROR findings support an aggressive treatment approach in patients with either SLN micrometastases or ITCs.

## 4. Completion ALND and Micrometastases

Many investigators have studied the incidence of non-SLN involvement in patients with SLN micrometastases to define which patients may need further axillary treatment. Wada and Imoto. collected 22 studies from 1999 until 2006 referring to the frequency of SLN micrometastases in patients with breast cancer and the prevalence of non-SLN involvement in those patients after ALND [47]. The frequency of SLN micrometastases was 38% with non-SLN micrometastases ranging from 0 to 57%. Additionally, a wide range of non-SLN macrometastases was found (0–18%). Because the prevalence of non-SLN micrometastases was low, the prognostic impact was unclear. The wide range of results arose from the different numbers of patients involved, variations in number of pathological sections examined, and differences in tumour stage and grade.

Results of studies in which patients with micrometastases in SNB and who were not treated by completion axillary node clearance are summarised in Table 3 [38, 48–54]. Most of the studies had small numbers and relatively short followup and tended to conclude that there was no benefit from completion axillary node clearance. The largest study; however, found a significantly worse disease-free survival for women with micrometastases who did not undergo cALND. [38].

De Boer et al. conducted a systematic review of 58 studies conducted from 1977 to 2008 included 297,533 patients, aiming to define the prognostic relevance of micrometastases and ITCs in patients with breast cancer [55]. Using random-effect meta-analysis they showed that the presence of ALN metastases <2 mm in diameter detected on single-section examination was associated with poorer overall survival. Moreover the presence of occult metastases on retrospective examination of ALN-negative patients by step sectioning and/or immunohistochemistry (*n* = 7740 patients) was associated with poorer 5-year disease-free and overall survival. Outcomes from sentinel lymph node biopsy studies were not assessable due to small patient groups and short followup.

The International Breast Cancer Study Group trial IBCSG-23-01 is a randomised multicentre study designed to determine the significance of minimal LN metastasis in patients with breast cancer [56]. The trial was initiated in April 2001, and it compares survival between patients with SLN micrometastases who undergo SLNB alone with those who receive cALND.

## 5. Completion Axillary Lymph Node Dissection in SLN Macrometastases

It is known that the extent of macrometastases in SLN is strongly correlated with non-SLN involvement. The long-term effect of the residual axillary disease in the sentinel-lymph-node-positive patient on local and systemic recurrence has not been clearly defined for patients receiving modern radiotherapy and chemotherapy. Older studies of patients with symptomatic breast cancers have shown that inadequate axillary surgery does lead to reduced overall survival [57–60].

Recently, the first results of the multicentre Z0011 trial were published [61]. The study set out to randomise 1900 women with breast cancer and 1–5 involved SNLN to either cALND or observation. All had a lumpectomy and tangential breast irradiation, but systemic therapy was at the discretion of the treating centre. After a median followup of 6.3 years, the relapse-free survival for the ALND group was 82% compared with 84% for the observation group, and the overall survival was 92% in both groups. Unfortunately the trial stopped accrual after 891 cases had been entered which makes it underpowered to detect a 5% difference in outcome. An additional aspect to be considered is that the Guy's wide excision studies showed no difference between the wide excision group and the radical mastectomy group at 10 years whereas after 25 years there was a significantly worse relapse-free and overall survival in the wide excision group with inadequately treated axillae [58].

In a retrospective study, Takei et al. confirmed the importance of cALND in SLN-positive patients with high nuclear grade and hormone-negative breast cancer. It was noticed that of 459 patients with macrometastatic disease treated with cALND, after a median follow-up period of 34 months, the axillary recurrence rate was only 0.6% [62]. Bilimoria et al. studied a cohort of 403,167 patients with clinically node-negative breast cancer that underwent SLNB from the US National Cancer Data Base (1998–2005) [63]. Of the 97,314 (24%) patients identified with nodal metastases, 28% had no further surgical intervention in the axilla and 72% underwent cALND. After a median followup of 63 months, it was found that in all patients and separately in those with macroscopic and microscopic nodal disease, the unadjusted axillary recurrence rate and overall survival were comparable. After adjustment for clinicopathological differences, there was a trend towards a lower risk of axillary recurrence and death in patients with macroscopic nodal involvement undergoing cALND. For those with micrometastases, recurrence rates were similar in those undergoing either SLNB alone or cALND.

Of 26,986 patients with SLNB-positive breast cancer from the SEER database (surveillance, epidemiology, and end results), 16% had no further axillary treatment and 84% had cALND [64]. After a median followup of 50 months, although a higher rate of ipsilateral regional recurrence was noticed in patients who underwent SLNB alone, no statistically significant differences in overall survival (OS) between patients who underwent SLNB alone versus complete ALND were found. The investigators suggested that in patients with small, low-grade primary tumours, positive ER status, older age and who have received segmental mastectomy, cALND may be omitted [64]. 

Hwang et al. reviewed the outcome of 3,366 patients with invasive breast cancer who underwent SLNB from 1993 to 2005 [65]. Of 750 SLN-positive patients, 65%, 45.9%, and 34.2% were pN1, pN1mi, and pN0 (i+), respectively. Of these patients, 196 had no further axillary surgery due to clinician and patient preference. According to clinicopathological variables, adjuvant treatment was applied and locoregional and distant recurrence and survival were studied. After a median followup of 29.5 months, no patient had an axillary recurrence, one had supraclavicular lymph node recurrence, and three patients developed metastatic disease to the lung or bone. The median time to recurrence was 32 months. Notably the patients with distant metastases had T3 grade III invasive carcinoma. Despite the low axillary recurrence rate, authors suggested that it is not possible from these results to conclude definitively that cALND should be abandoned for these patients [65]. 

## 6. Predictive Models

Several factors correlated with the likelihood of additional non-SLN metastasis have been investigated in an effort to distinguish which patients could avoid extensive axillary surgery. Characteristics of the primary tumour, such as size [40, 66], grade [67], hormone receptor and HER2 profile [67], tumour type [67], multifocality, mean proliferative fraction, and lymphovascular invasion [68, 69], have all been studied. Additional features of the involved SLNs, such as size of metastases [40], number of positive SLNs [40], ratio of positive to resected SLNs, and the extracapsular spread have been also examined [21, 61, 70–72]. Particularly patients with minimal SLN metastases are at a significantly lower risk to have further non-SLN invasion than those with SLN macrometastases (13–24% versus 45–79%) [61]. However, none of these characteristics individually can determine a subset of patients for whom ALND is unnecessary. Molecular profiling of metastatic foci different from the primary tumour could be used as indicator for the selection of patients who might benefit of completion axillary dissection [73]. The most important prognostic factors for the presence of non-SLN metastases in patients with minimal SLN involvement are presented in Table 4.

Several mathematical models have been developed to predict the risk of non-SLN involvement in patients with SLN-positive breast cancer [74]. These include four nomograms: the Memorial Sloan-Kettering Cancer Center (MSKCC) [75], (https://www.mskcc.org/mskcc/html/15938.cfm), Mayo [76] (https://www.mayoclinic.org/breast-cancer/sentinelbiopsy.html), Cambridge [77], and Stanford [70] (https://www3-hrpdcc.stanford.edu/nsln-calculator/). There are three scoring systems, the Tenon, MDA, and Saidi, and two recursive partitioning (RP) tools developed by Kohrt et al. [70]. 

The Institut Curie studied 588 consecutive patients with positive SLNs who underwent ALND to compare the actual rate of non-SLN metastases with those predicted by Breast Cancer Nomogram of Memorial Sloan-Kettering Cancer Center (MSKCC). While the predicted rate in non-SLN macrometastases was relatively accurate, when the nomogram was applied to the 213 SLNs that contained only micrometastases, the predicted rate 5–9% was far away from the actual rate 44% of non-SLN micrometastases detected by IHC. Consequently, the authors concluded that a different predictive model should be created for patients with micrometastases.

Molecular tests based on technology such as Oncotype Dx (Genomic Health, Redwood City, Calif, USA) or other multigene arrays developed prognostic and predictive markers aiming to personalize surgical and adjuvant treatment of early breast cancer [4]. An accurate, intraoperative sentinel lymph node test probably could help in avoidance of delayed axillary dissections. Molecular tests may be proved more sensitive than current intraoperative tests but have not yet been validated.

## 7. One-Step Nucleic Acid Amplification (OSNA)

OSNA is an automated assay for the detection of cytokeratin message, CK19 mRNA, present in approximately 98% of breast cancers [78]. It provides an opportunity to make an intraoperative diagnosis of sentinel node involvement within 30 minutes, avoiding frozen section and allowing a one-stage procedure. Larger verification studies in which half of the bisected sentinel node was sent for histology and the other half homogenised for OSNA are shown in Table 5 [71, 72, 79–81]. The usual reason for discordance between histopathology and OSNA was an uneven distribution of nodal metastases (tissue allocation bias). This problem is abolished when the entire node is subjected to OSNA.

The studies indicate a good concordance between results of histopathology and OSNA. Indeed in a study conducted by Osaka, comparing OSNA with frozen section, the former was more sensitive, increasing the positive sentinel node rate by 30% [82]. It is likely that in time OSNA will replace histopathological examination of sentinel lymph nodes because of its ease, accuracy, and potential for enabling almost all patients to have a one-stage operation for early breast cancer.

## 8. Conclusions 

Recent studies aiming to determine if cALND is beneficial in patients with SLN-positive breast cancer and even more in patients with minimal SLN involvement have reached contrary conclusions. Limitations in the published studies include the methods of pathological evaluation of lymph nodes and that the number of additional non-SLN-positive nodes is usually not known. Detection rate of micrometastases and ITCs depends on histopathological technique and protocol. Thus lymph node step sectioning and IHC lead to increased identification of minimal metastases upstaging 9% to 25% of patients who initially were considered node negative [83, 84] and did not have further axillary surgery. 

The different rates of recurrence may be due to the molecular type of cancers and so that patients with SLN metastases may also have different risks of metastatic involvement [40]. Finally it is possible that not all minor tumour foci in axillary lymph nodes progress to local recurrences. According to Al-Hajj et al., only a minority of cancer cells potentially give metastases and most ITCs are not viable and do not have the ability to form new tumours [85]. There may be two different breast cancer cell populations, true stem cells that have the capacity to develop metastases and the nonstem cells that never grow and are finally destroyed [86]. 

At the level of everyday clinical practice, with both promising and disappointing results of the published studies, most breast surgeons will hardly ever take the risk of avoiding completion axillary dissection in breast cancer patients even with minimal sentinel lymph node metastases. Many are seeking to find a balance between the needs of the majority to have minimal axillary surgery with minimal postoperative morbidity against the possibility that a minority will suffer relapse, morbidity, and possible increased mortality from undertreatment. Results from ongoing phase III trials will perhaps provide new guidelines for the treatment of patients with micrometastases or ITCs. A predictive model which estimates accurately the likelihood of additional disease in the axilla might help tailor surgical therapy to the needs of the individual patient and identify those most likely to benefit from completion or ALND. Genetic assays defining prognostic markers and new intraoperative tests detecting accurately SLN involvement will help in early therapeutic decision making in the future. It is important that premature decisions to restrict axillary surgery are not made on a basis of early results from underpowered clinical trials.

## Figures and Tables

**Table 1:** Recommendations of SLNB, ALND, and treatment after SLNB.

SLNB	ALND	Post-SLNB	
T1, T2 tumour	T3, T4 tumours	SLNB +ve	ALND
Multicentric tumour	Inflammatory carcinoma	SLNB −ve	Observe
DCIS for mastectomy	Suspicious axillary node	Micromets	ALND
DCIS > 5 cm	Pregnancy	ITC	Observe
Older patient	Prior axillary surgery	SLNB +ve, 1-2 nodes, T1, T2	Observe*
Preneoadjuvant			

DCIS: ductal carcinoma-in-situ; SNB: sentinel lymph node biopsy; ALND: axillary lymph node dissection.

*Recommendation of the German guidelines.

**Table 2:** Incidence and prognostic impact of ITCs in sentinel node biopsies.

Author	Total	ITC (%)	Outcome
Herbert et al. [37]	514	16 (3%)	No effect
Reed et al. [38]	1255	25 (2%)	No effect
De Boer et al. [39]	2707	819	HR 1.5 (No adjuvant versus adjuvant)
Barbosa et al. [40]	1000	43 (4%)	No effect
Andersson et al. [41]	3369	107 (3%)	No effect
Leidenius et al. [42]	1390	63 (5%)	Reduced 5-year survival
Maaskant-Braat et al. [43]	6803	126 (2%)	No effect

**Table 3:** Studies of patients with micrometastases not treated by completion mastectomy.

Author	Total	Follow-up (months)	Outcome
Fan et al. [48]	27	17	1 recurrence
Nagashima et al. [49]	19	24	1 recurrence
Yegiyants et al. [50]	33	84	1 recurrence
Fournier et al. [51]	16	30	No recurrence
Langer et al. [52]	27	77	No recurrence
Meretoja et al. [53]	48	37	3 recurrences, 1 death
Pernas et al. [54]	45	60	1 recurrence
Reed et al. [38]	57	59	Significantly reduced disease-free interval

**Table 4:** Major prognostic factors for non-SLN metastases in patients with minimal SLN metastases.

Feature	Author
Lymphovascular invasion	Mittendorf et al. [67], Van Deurzen et al. [66], Viale et al. [69], Jinno et al. [68]

Size of SLN metastases	Barbosa et al. [40], Van Deurzen et al. [66], Viale et al. [69]

Primary tumour size	Barbosa et al. [40], Van Deurzen et al. [66]

Lobular histology	Mittendorf et al. [67]

Number of positive SLN	Barbosa et al. [40], Viale et al. [69], Jinno et al. [68]

**Table 5:** Validation studies comparing OSNA with histopathology.

Author	Total	Concordance	Sensitivity	Specificity
Tsujimoto et al. [71]	101	98%	91%	100%
Schem et al. [72]	93	92%	98%	91%
Tamaki et al. [79]	185	93%	88%	94%
Snook et al. [80]	204	96%	92%	97%
Feldman et al. [81]	498	96%	78%	96%
